# Supplementary material for: Previous infection with seasonal coronaviruses does not protect male Syrian hamsters from challenge with SARS-CoV-2
Source: Nat Commun. 2023 Sep 26;14:5990. doi: 10.1038/s41467-023-41761-1 (PMC10522707; doi:10.1038/s41467-023-41761-1)
Supplement: Supplementary file 1 — Supplementary Information [file 41467_2023_41761_MOESM1_ESM.pdf]

Supplementary Figure 1

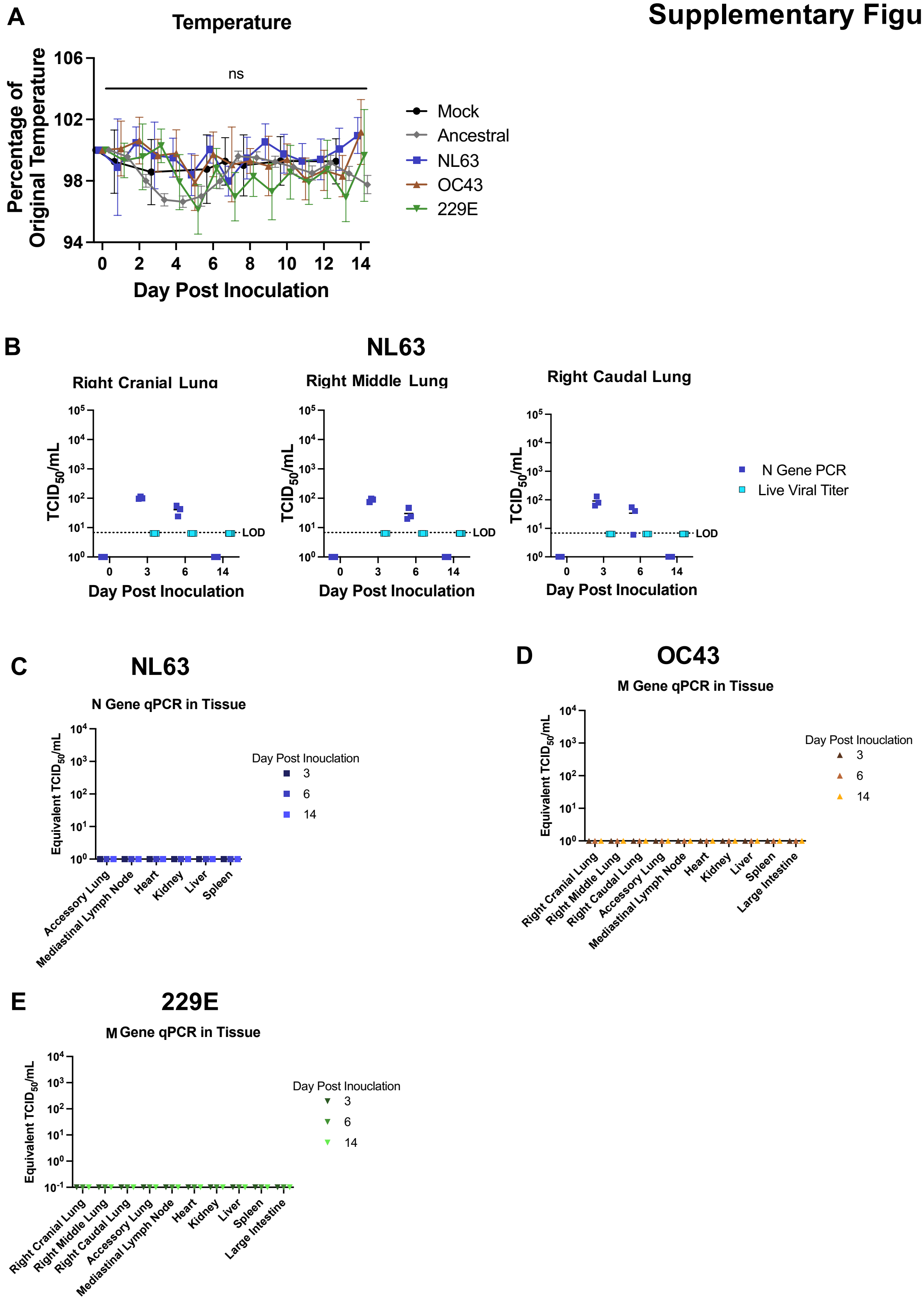

Supplementary Fig. 1. Temperature, viral RNA, and live virus analysis following seasonal coronavirus inoculation in Syrian hamsters. Temperature was recorded daily following inoculation in Syrian hamsters with 229E, NL63, and OC43 (**A**)**.** Temperature was compared to control mock inoculated animals and animals inoculated with SARS-CoV-2 ancestral virus. NL63 viral RNA was present in the lower respiratory tract of inoculated hamsters as determined by qRT-PCR (**B**). No corresponding live virus was detected in these tissues. qRT-PCR was used to assess the presence of viral RNA in the extrapulmonary tissues of NL63 inoculated animals (**C**) as well as all tissues collected at necropsy on day 3, 6, and 14 ppi for OC43 (**D**) and 229E (**E**) inoculated hamsters. LOD is the limit of detection. N is equal to or greater than 3 for all timepoints. ns indicates no significant differences.

Supplementary Figure 2

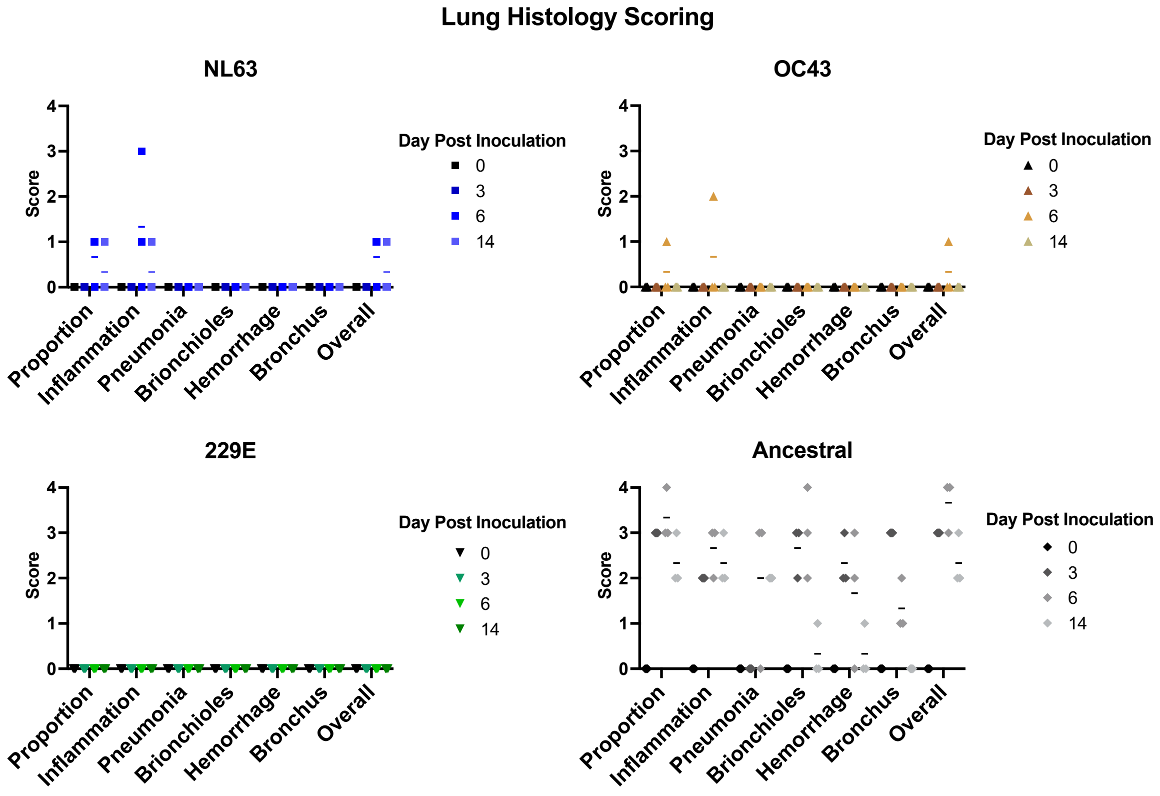

Supplementary Fig. 2. Inoculation with NL63 or OC43 led to minimal changes in histological scoring in the lung compared to inoculation with ancestral SARS-CoV-2 lineage B virus. Collected lungs from each group post inoculation were processed for histological assessment. Lungs were formalin perfused, sectioned, mounted, and H&E stained followed by visualization and analysis by a board-certified pathologist. Lungs were given a score ranging from 0 to 4 corresponding to seven categories. Proportion describes the percentage of parenchyma affected where 1= <25%; 2= 26-50%; 3= 51-75% 4= 76-100%. Inflammation indicated the density of the inflammatory infiltrate in affected areas. Pneumonia details the extent of hypertrophy and/or hyperplasia of alveolar pneumocytes. Bronchioles scores corresponds epithelial lesions in small bronchioles (extending into terminal bronchioles). Hemorrhage rates intra-alveolar hemorrhage. Bronchus focuses on intrapulmonary portion of a large bronchus and its larger tributaries including infiltration of inflammatory cells in wall. Overall gives a summary score taking all factors into account. 0, absent (no lesion); 1, slight or questionable; 2, clearly present, but not conspicuously so; 3, marked; 4, severe.

Supplementary Figure 3

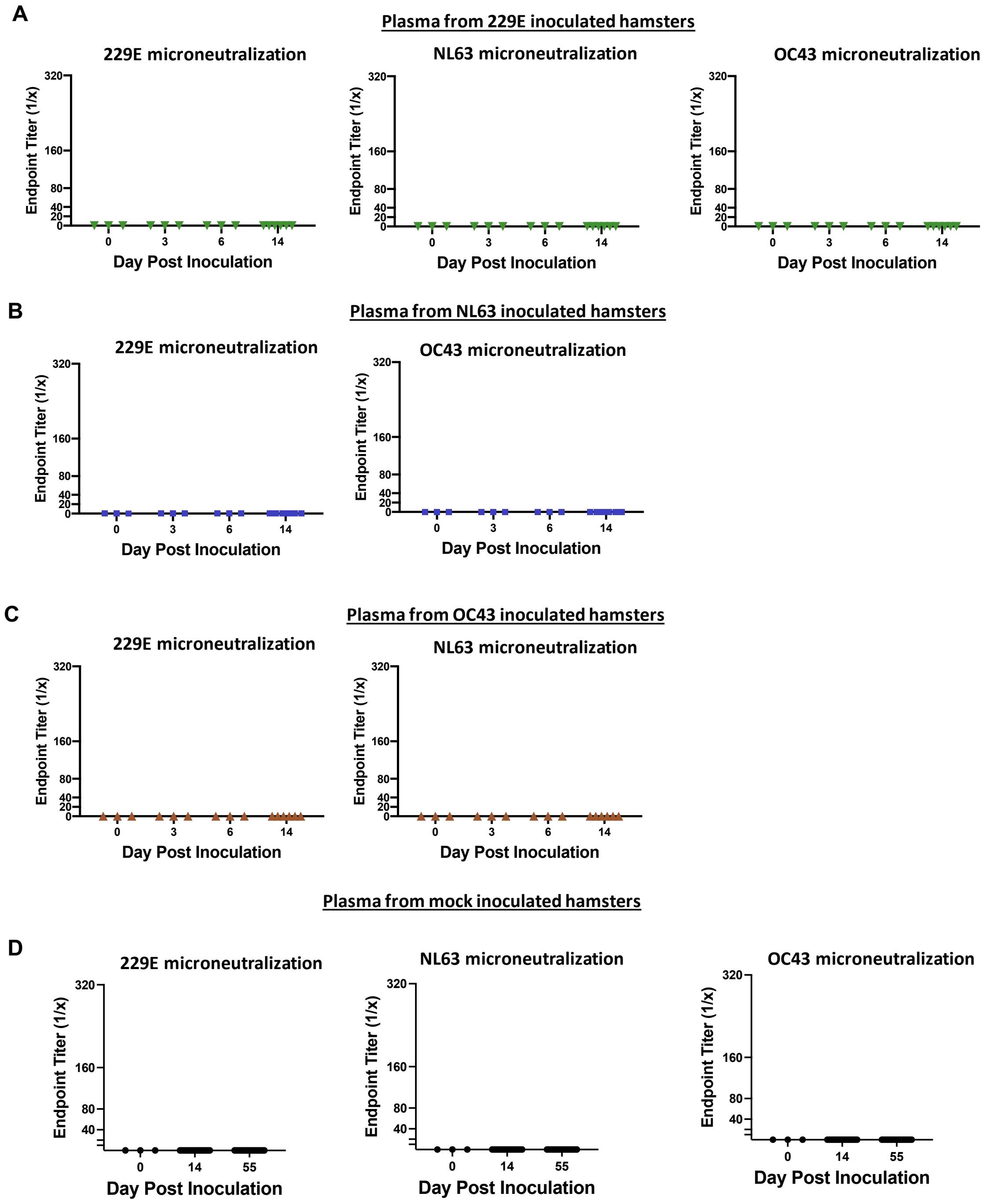

Supplementary Fig. 3. Cross-neutralizing antibodies were not elicited in 229E, NL63, or OC43 inoculated hamsters. Plasma collected from 229E (**A**), NL63 (**B**), OC43 (**C**), and mock (**D**) inoculated animals on days 0, 3, 6, and 14 ppi were used for microneutralization assays against seasonal coronaviruses.

Supplementary Figure 4

Host Gene Expression qPCR
Nasal Turbinates

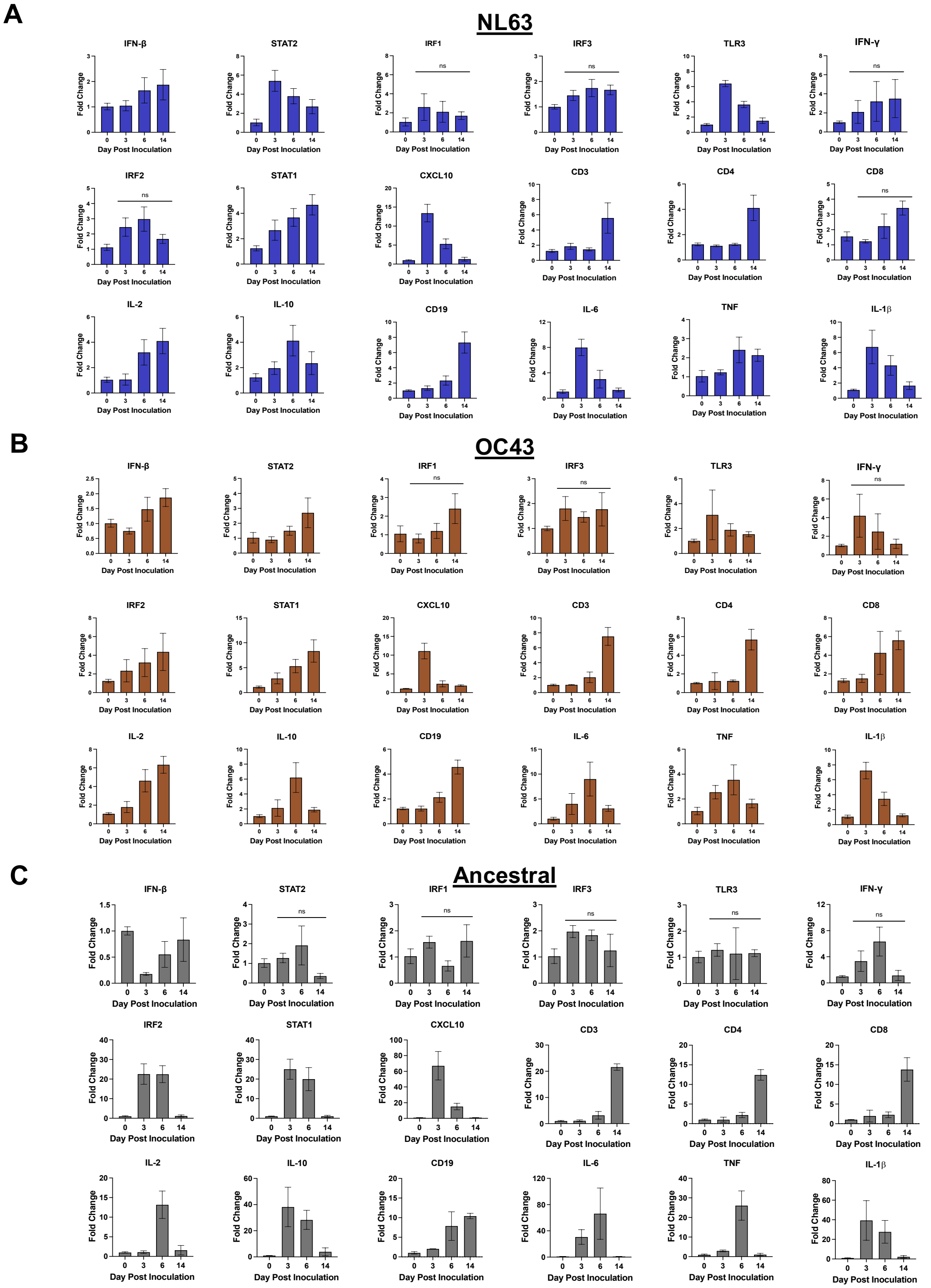

Supplementary Fig. 4. Inoculation with seasonal coronaviruses NL63 and OC43 led to intact type I interferon and diminished inflammatory gene expression in the nasal turbinates compared to ancestral virus. qRT-PCR was performed on RNA extracted from the nasal turbinates following inoculation with NL63 (**A**), OC43 (**B**) or ancestral SARS-CoV-2 (**C**) in Syrian hamsters. Genes associated with the type I interferon response (IFN-β, STAT2, IRF1, IRF3 and TLR3), the type II/general interferon response (IFN-γ, IRF2, STAT1, and CXCL10), T cells (CD3, CD4, CD8A, IL-2, IL-10), B Cells (CD19) and inflammatory response (IL-6, TNF, and IL-1β) were analyzed. Primers were designed or acquired specific to hamster genes (Table 2). Fold-change was calculated via ΔΔCt against baseline (Day 0) with BACT as the housekeeping gene. At least three animals were analyzed for each timepoint. * represents a significant difference from baseline (day 0).

Supplementary Figure 5

Host Gene Expression qPCR
Right Cranial Lung

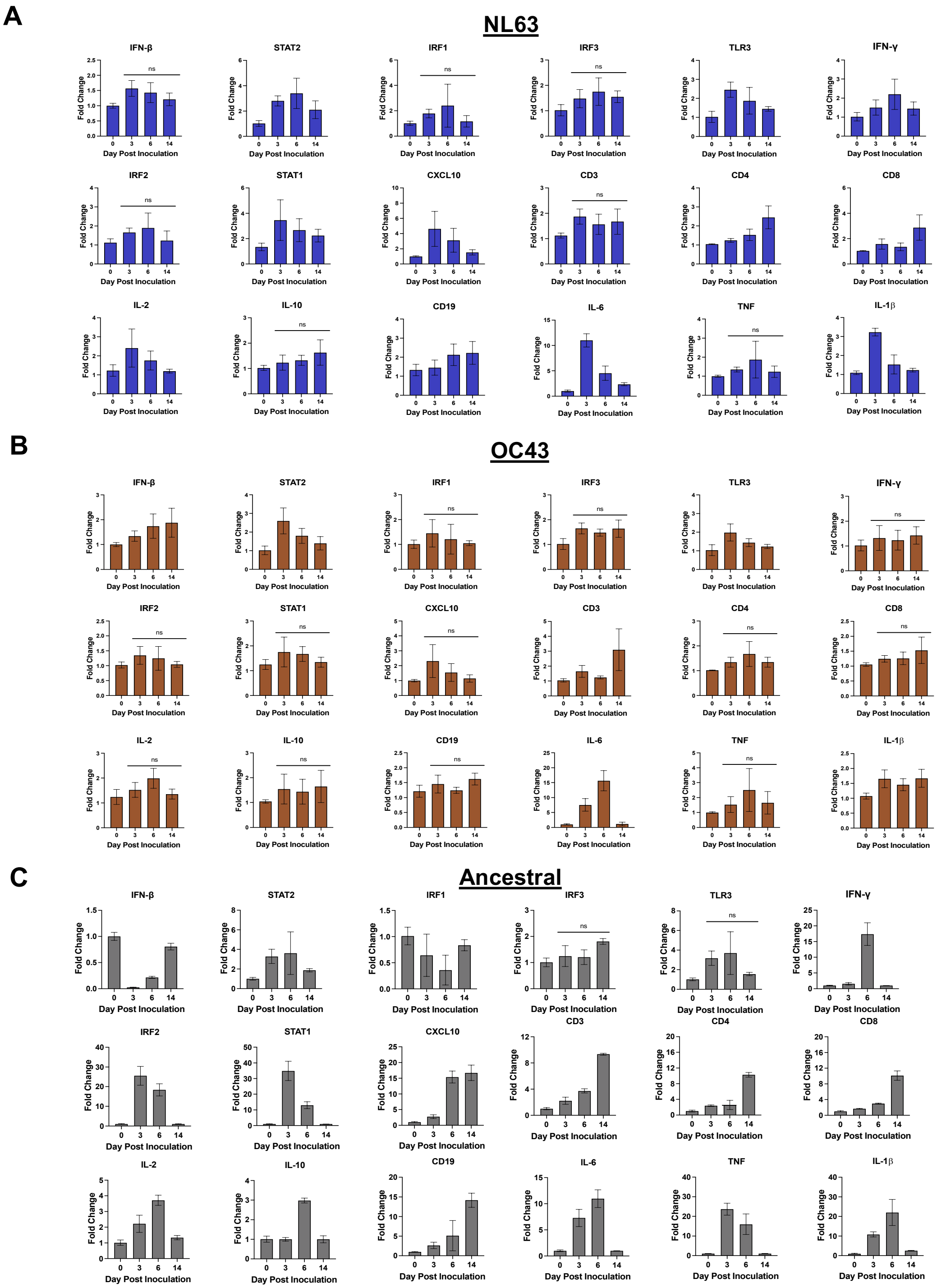

Supplementary Fig. 5. Infection with seasonal coronaviruses NL63 and OC43 did not lead to downregulation of type I interferon responses or upregulate inflammatory genes in the lung. qRT-PCR was performed on RNA extracted from the right cranial lung following inoculation with NL63 (**A**), OC43 (**B**) or ancestral SARS-CoV-2 (**C**) in Syrian hamsters. Genes associated with the type I interferon response (IFN-β, STAT2, IRF1, IRF3 and TLR3), the type II/general interferon response (IFN-γ, IRF2, STAT1, and CXCL10), T cells (CD3, CD4, CD8A, IL-2, IL-10), B Cells (CD19) and inflammatory response (IL-6, TNF, and IL-1β) were analyzed). Primers were designed or acquired specific to hamster genes (Table 2). Fold-change was calculated via ΔΔCt against baseline (Day 0) with BACT as the housekeeping gene. At least three animals were analyzed for each timepoint. * represents a significant difference from baseline (day 0).

Supplementary Figure 6

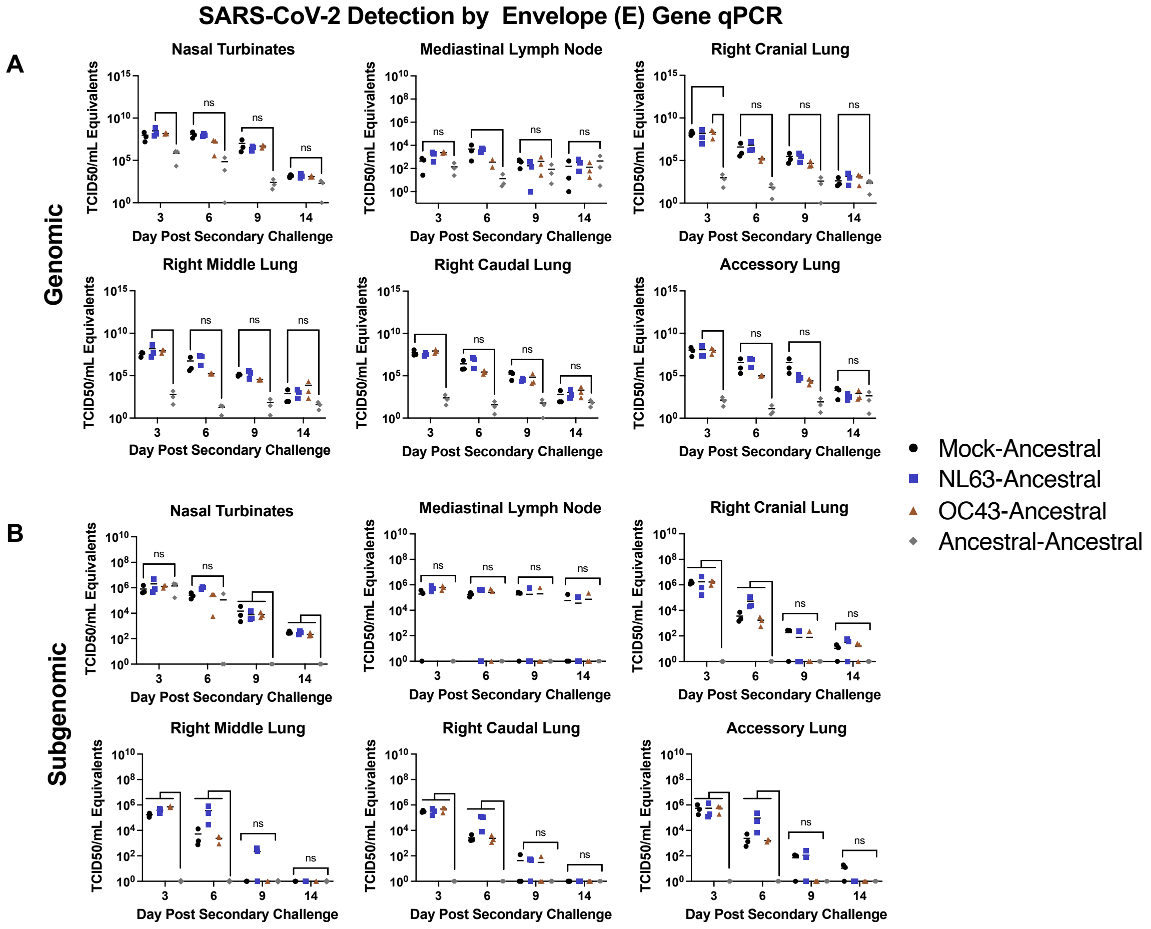

Supplementary Fig. 6. viral RNA was decreased in tissues following SARS-CoV-2 ancestral virus secondary challenge in ancestral virus inoculated hamsters compared to seasonal coronavirus inoculated hamsters. RNA was extracted from respiratory tissues and the mediastinal lymph node following SARS-CoV-2 ancestral virus secondary challenge in NL63, OC43, SARS-CoV-2 ancestral virus, and mock inoculated hamsters. Genomic viral RNA (**A**) and subgenomic viral RNA (**B**) were assessed by qRT-PCR. * indicates a p-value less <0.05 determined by ANOVA comparing hamsters on the days post inoculation to baseline (day 0).

Supplementary Figure 7

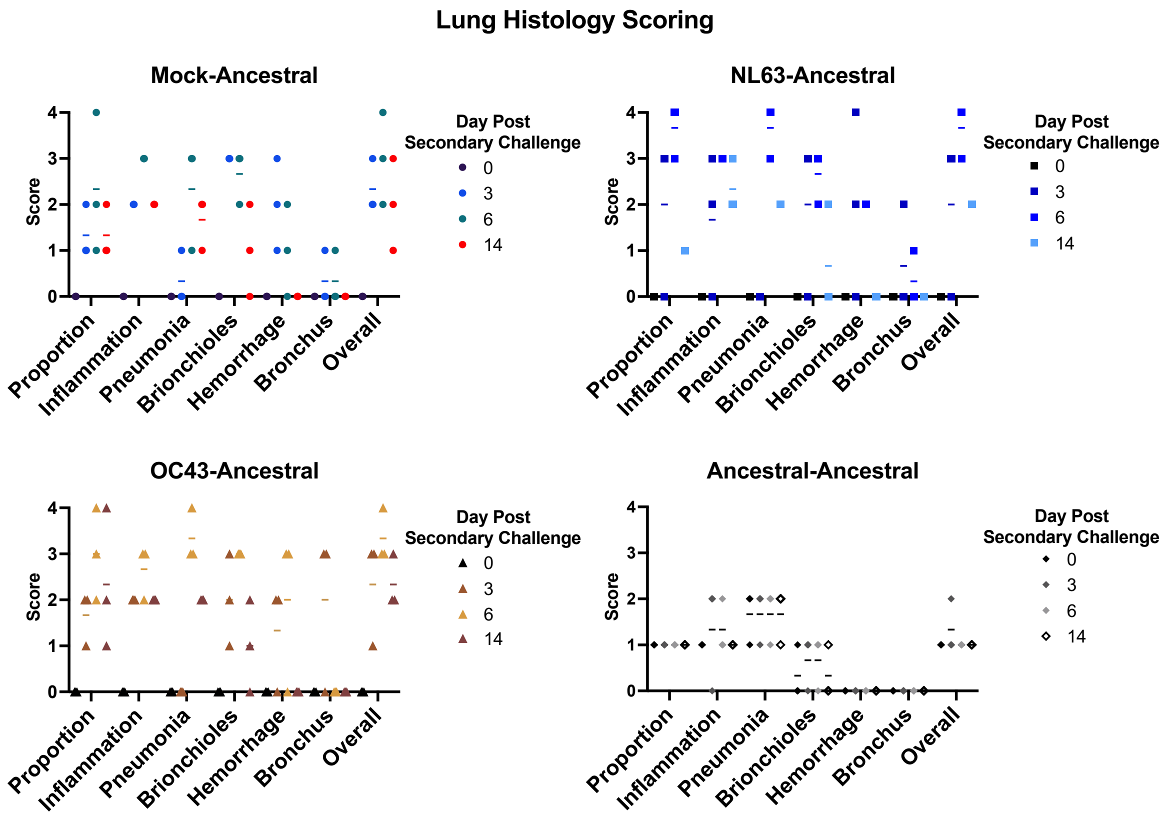

Supplementary Fig. 7. Inoculation with seasonal coronaviruses did not protect against lung damage resulting from a secondary inoculation with ancestral virus. Collected lungs from each group post inoculation were processed for histological assessment. Lungs were formalin perfused, sectioned, mounted, and H&E stained followed by visualization and analysis by a board-certified pathologist. Lungs were given a score ranging from 0 to 4 corresponding to seven categories. Proportion describes the percentage of parenchyma affected where 1= <25%; 2= 26-50%; 3= 51-75% 4= 76-100%. Inflammation indicated the density of the inflammatory infiltrate in affected areas. Pneumonia details the extent of hypertrophy and/or hyperplasia of alveolar pneumocytes. Bronchioles scores corresponds epithelial lesions in small bronchioles (extending into terminal bronchioles). Hemorrhage rates intraalveolar hemorrhage. Bronchus focuses on intrapulmonary portion of a large bronchus and its larger tributaries including infiltration of inflammatory cells in wall. Overall gives a summary score taking all factors into account. 0, absent (no lesion); 1, slight or questionable; 2, clearly present, but not conspicuously so; 3, marked; 4, severe.

Supplementary Figure 8

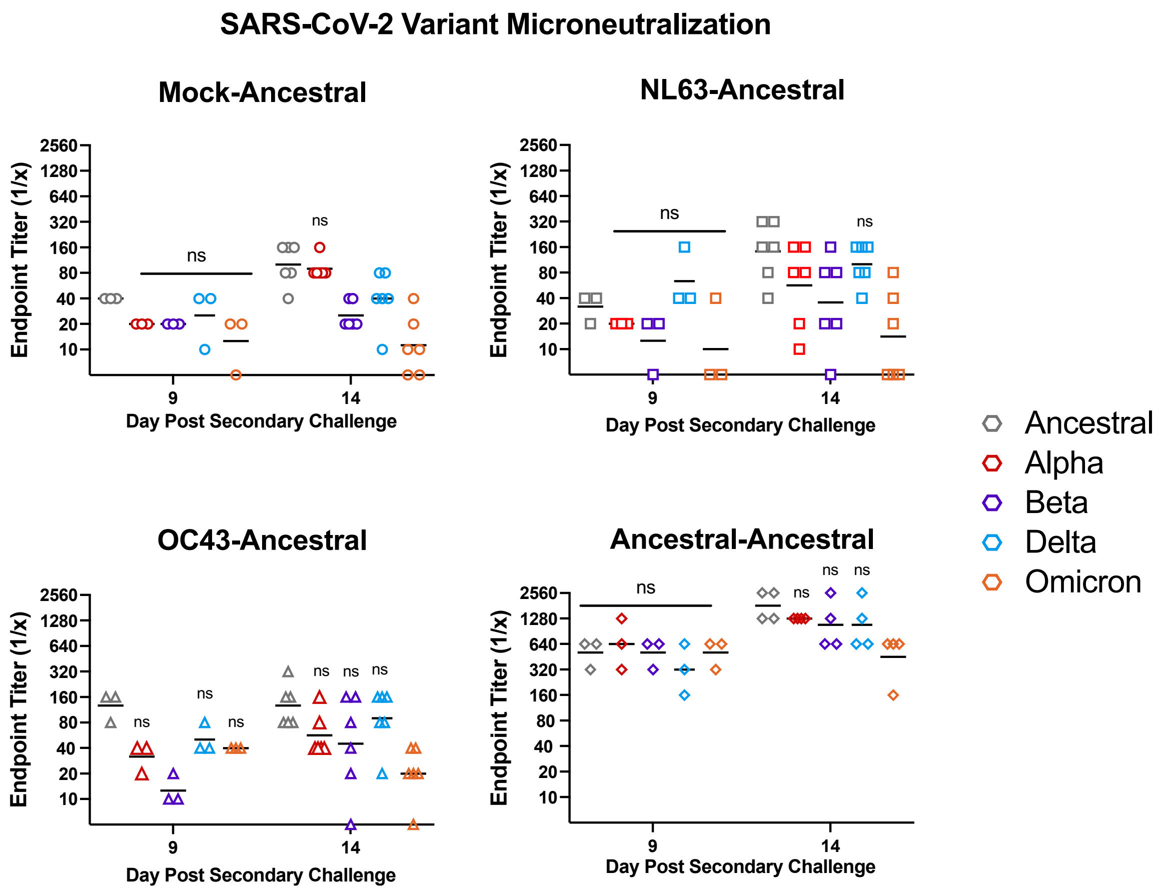

Supplementary Fig. 8. Seasonal coronaviruses inoculated animals had higher titers of neutralizing antibodies against SARS-CoV-2 variants following secondary challenge. Following secondary challenge to establish Mock-Ancestral (top left), NL63-Ancestral (top right), OC43-Ancestral (bottom left), and Ancestral-Ancestral groups (bottom right), virus neutralizing antibodies were quantified against ancestral SARS-CoV-2 (grey) and the variants Alpha (red), Beta (purple), Delta (light blue) and Omicron (orange) to determine the potential for cross-neutralization. Line represents geometric mean. * indicates a p-value less <0.05 determined by ANOVA comparing variant titers to the challenge virus, ancestral SARS-CoV-2, titers.

Supplementary Figure 9

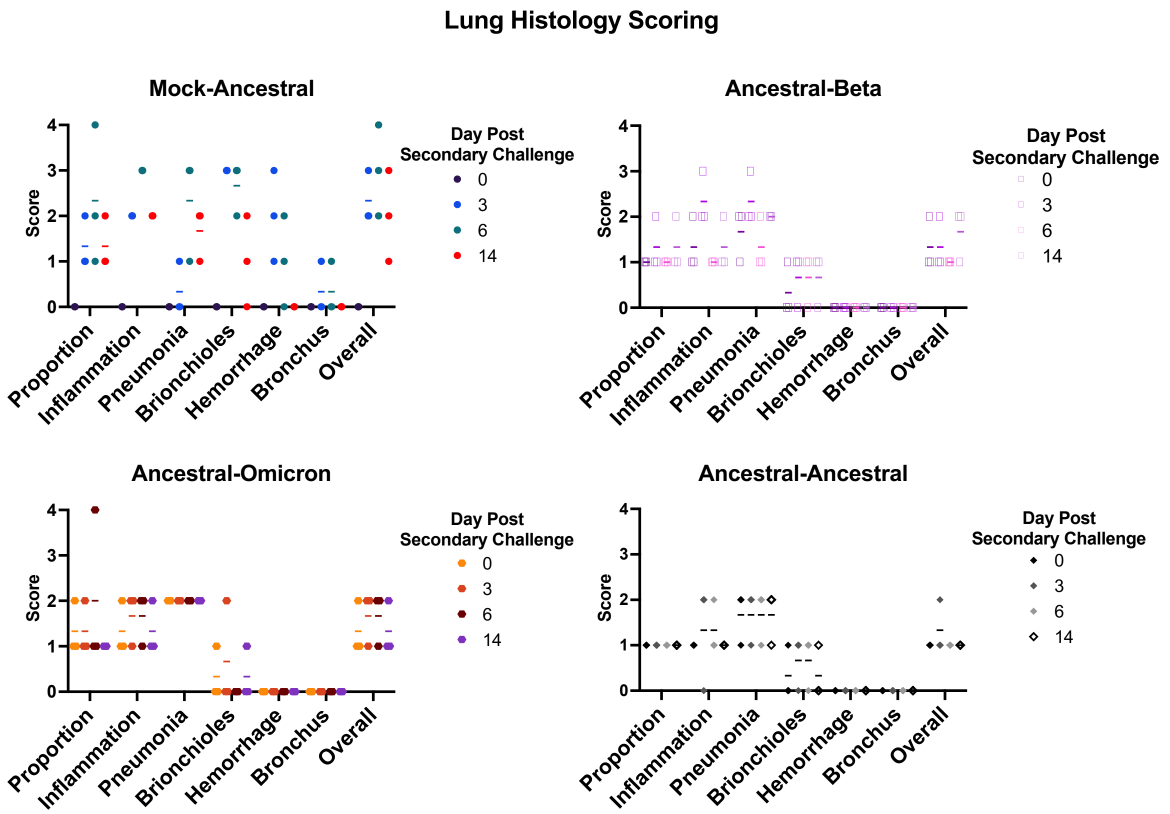

Supplementary Fig. 9. Animals with a primary inoculation of lineage B virus had decreased lung damage following a secondary inoculation with Beta and Omicron as determined by histological scoring. Collected lungs from each group post inoculation were processed for histological assessment. Lungs were formalin perfused, sectioned, mounted, and H&E stained followed by visualization and analysis by a board-certified pathologist. Lungs were given a score ranging from 0 to 4 corresponding to seven categories. Proportion describes the percentage of parenchyma affected where 1= <25%; 2= 26-50%; 3= 51-75% 4= 76-100%. Inflammation indicated the density of the inflammatory infiltrate in affected areas. Pneumonia details the extent of hypertrophy and/or hyperplasia of alveolar pneumocytes. Bronchioles scores corresponds epithelial lesions in small bronchioles (extending into terminal bronchioles). Hemorrhage rates intra-alveolar hemorrhage. Bronchus focuses on intrapulmonary portion of a large bronchus and its larger tributaries including infiltration of inflammatory cells in wall. Overall gives a summary score taking all factors into account. 0, absent (no lesion); 1, slight or questionable; 2, clearly present, but not conspicuously so; 3, marked; 4, severe.

Supplementary Figure 10

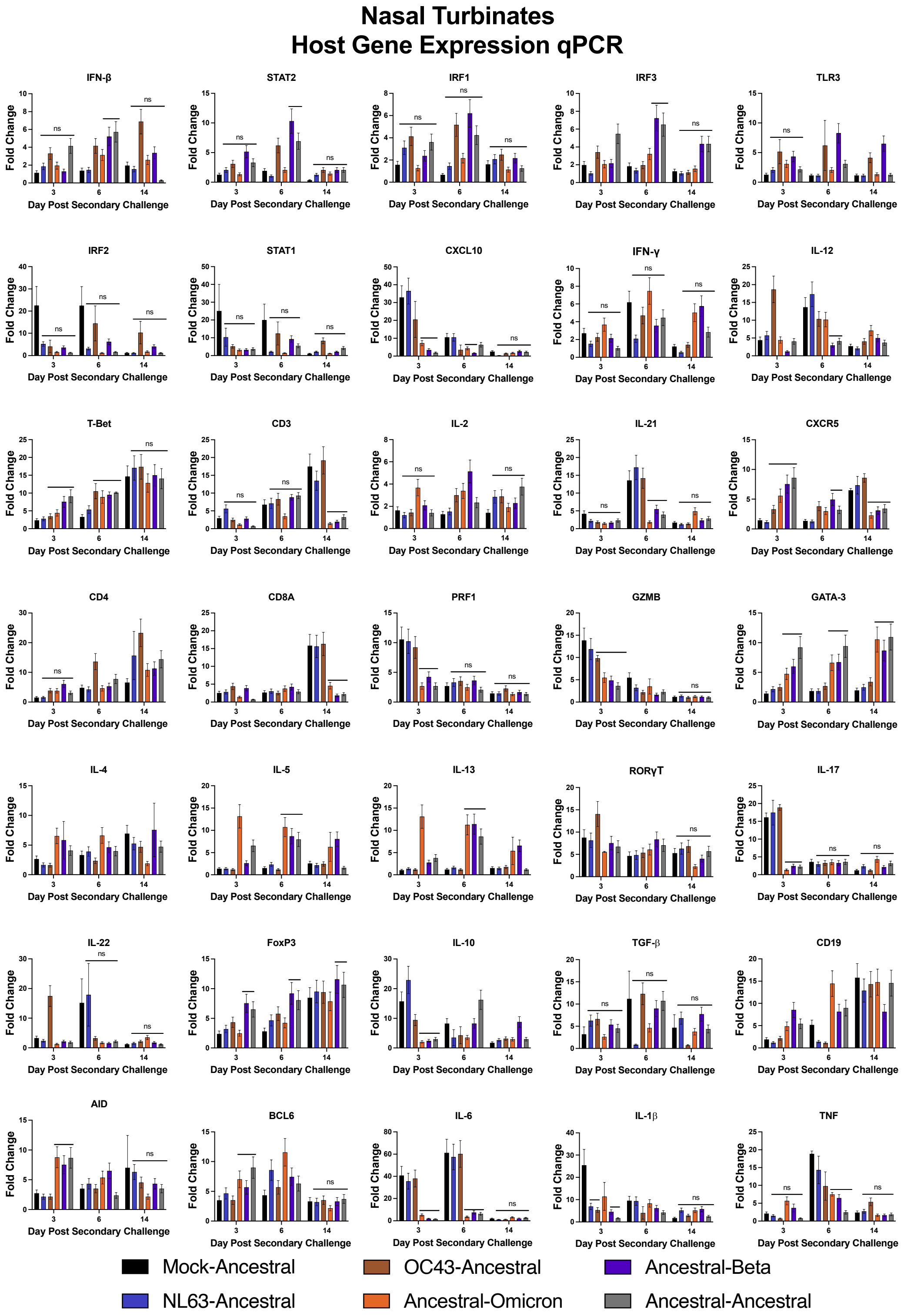

Supplementary Fig. 10. Bar Graph Representation of Host Gene Expression qPCR in Nasal Turbinates (Figure 7A). qRT-PCR was performed on RNA extracted from nasal turbinate following secondary challenge in coronavirus inoculated hamsters. Primers were designed or acquired specific to hamster genes (Table 2). Fold-change was calculated via ΔΔCt against baseline (Day 0) with BACT as the housekeeping gene. At least three animals were analyzed for each timepoint. Statistical differences from Mock-Ancestral are represented by asterisk.

Supplementary Figure 11

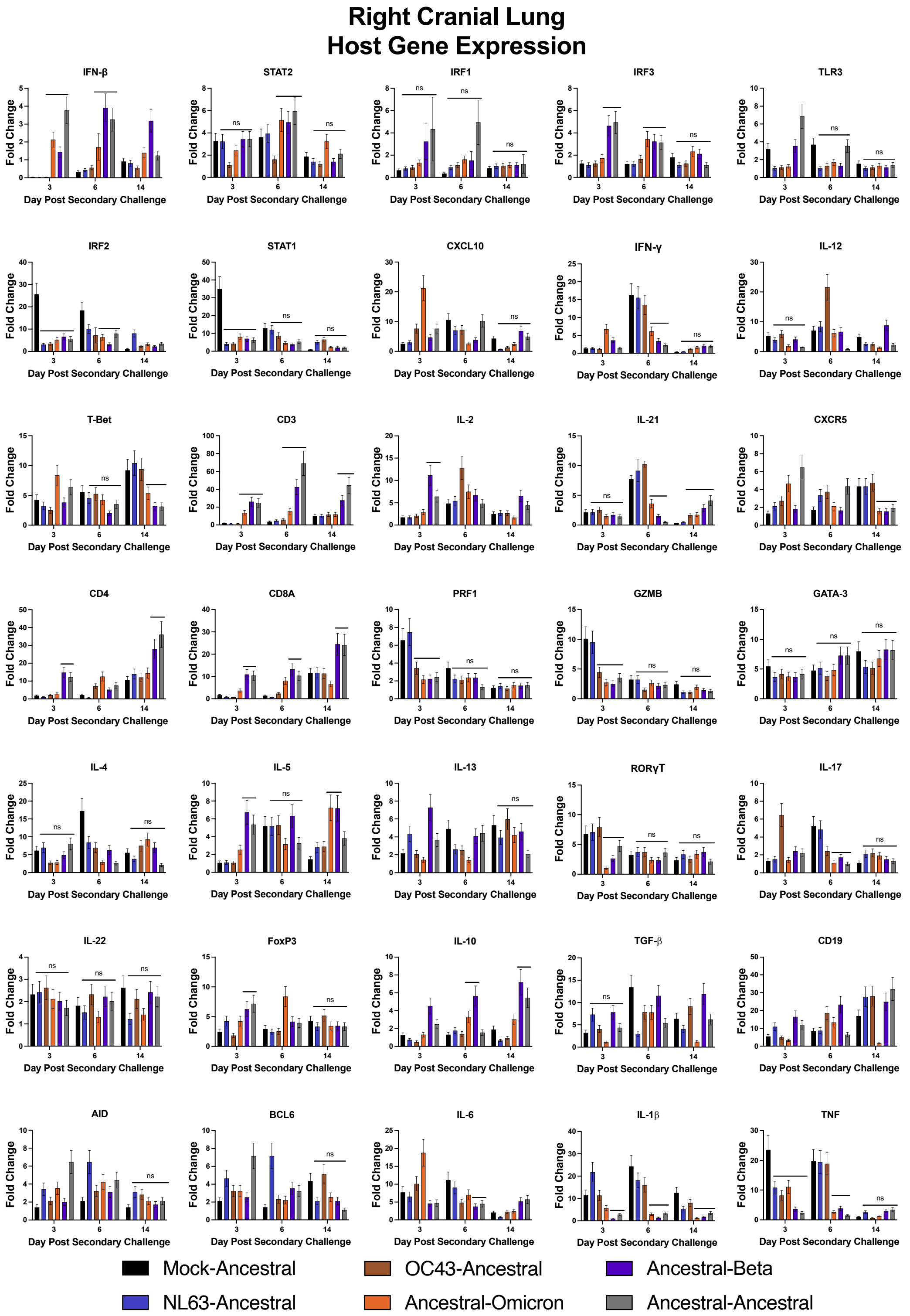

Supplementary Fig. 11. Bar Graph Representation of Host Gene Expression qPCR in Right Cranial Lung (Figure 7B). qRT-PCR was performed on RNA extracted from right cranial lung following secondary challenge in coronavirus inoculated hamsters. Primers were designed or acquired specific to hamster genes (Table 2). Fold-change was calculated via ΔΔCt against baseline (Day 0) with BACT as the housekeeping gene. At least three animals were analyzed for each timepoint. Statistical differences from Mock-Ancestral are represented by asterisk.

Supplementary Figure 12

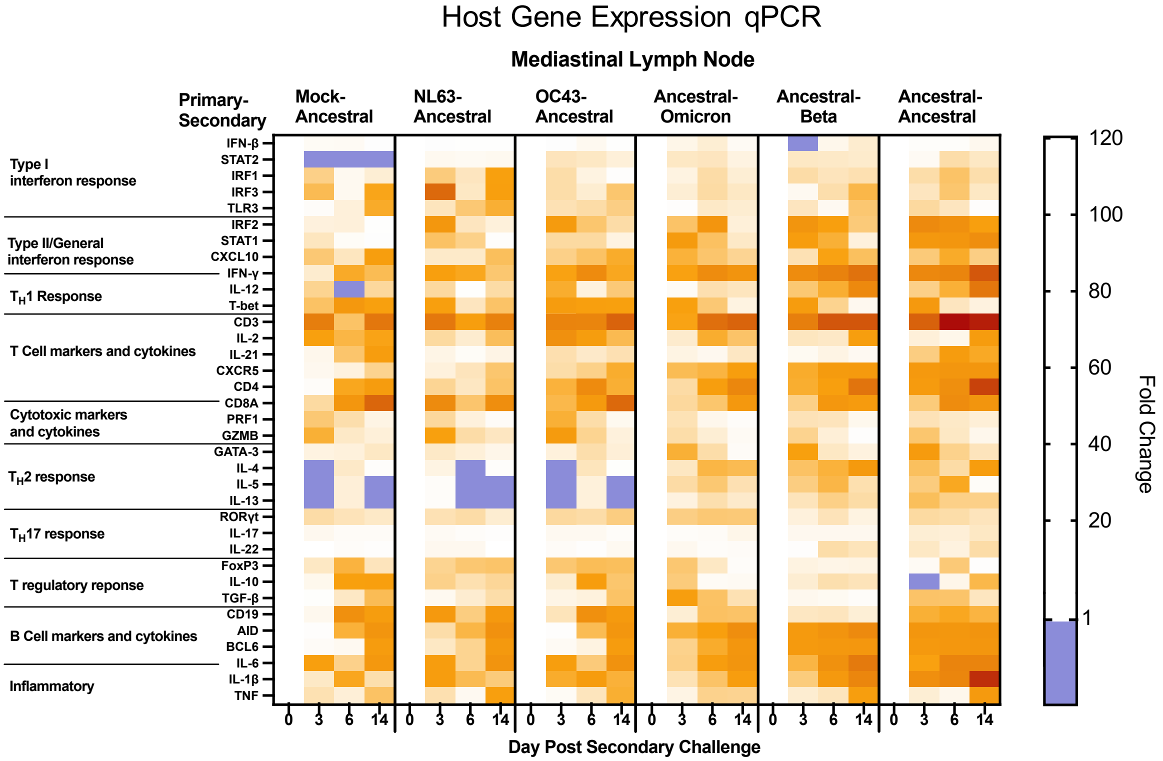

Supplementary Fig. 12. Complete gene expression analysis in the mediastinal lymph node at secondary challenge varies with antigenic distance between inoculating and secondary challenge virus visualized as a heatmap. qRT-PCR was performed on RNA extracted from the mediastinal lymph node following secondary challenge in coronavirus inoculated hamsters. Gene regulation was analyzed according to response type and results were displayed as a heat map according to fold regulation. Groups are arranged according to antigenic distance between the inoculating and secondary challenge virus. Genes were organized by type I interferon response (IFN-β, STAT2, IRF1, IRF3 and TLR3), type II/general interferon response (IRF2, STAT1, CXCL10 and IFN-γ), TH1 response (IFN-γ, IL-12 and T-bet), T cell makers and cytokines (CD3, IL-2, IL-21, CXCR5, CD4 and CD8A), cytotoxic markers and cytokines (CD8A, PRF1 and GZMB), TH2 response (GATA-3, IL-4, IL-5 and IL-13), T regulatory response (FoxP3, IL-10, and TGF-β), B cell markers and cytokines (CD19, AID, BCL6 and IL-6), and inflammatory cytokines (IL-6, IL-1β and TNF). Primers were designed or acquired specific to hamster genes (Table 2). Fold-change was calculated via ΔΔCt against baseline (Day 0) with BACT as the housekeeping gene. The legend depicts fold change in which upregulation (greater than 1) is represented ranging in from white (=1) to red. Any downregulation (less than 1) is represented in blue. At least three animals were analyzed for each timepoint. Significant difference from Mock-Ancestral animals can be found in Supplementary Table 6. Bar graphs of this data can be found in Supplementary Fig. 13.

Supplementary Figure 13

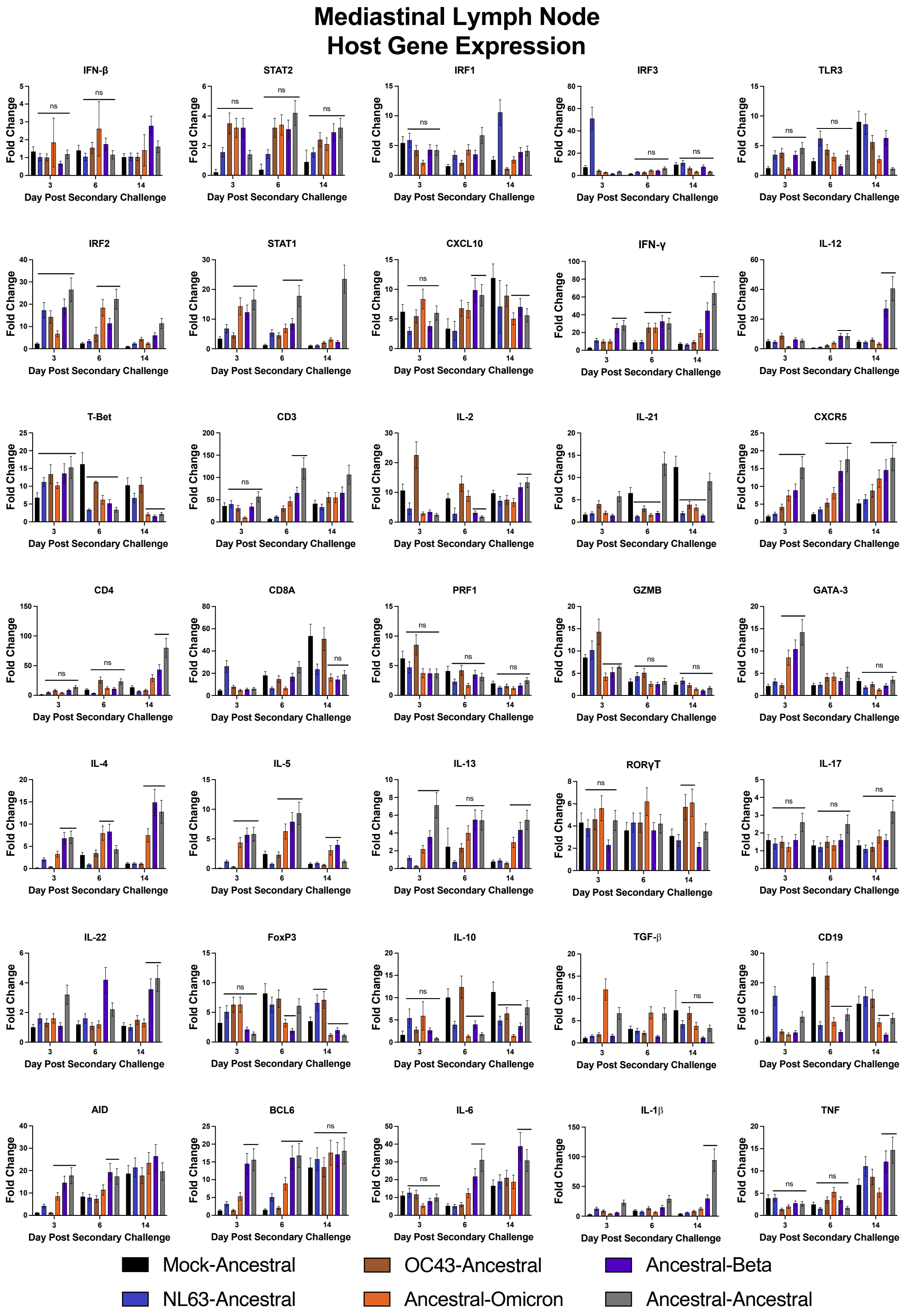

Supplementary Fig. 13. Bar Graph Representation of Host Gene Expression qPCR in Mediastinal Lymph Node (Supplementary Figure 12). qRT-PCR was performed on RNA extracted from mediastinal lymph node secondary challenge in coronavirus inoculated hamsters. Primers were designed or acquired specific to hamster genes (Table 2). Fold-change was calculated via ΔΔCt against baseline (Day 0) with BACT as the housekeeping gene. At least three animals were analyzed for each timepoint. Statistical differences from Mock-Ancestral are represented by asterisk.

Supplementary Figure 14

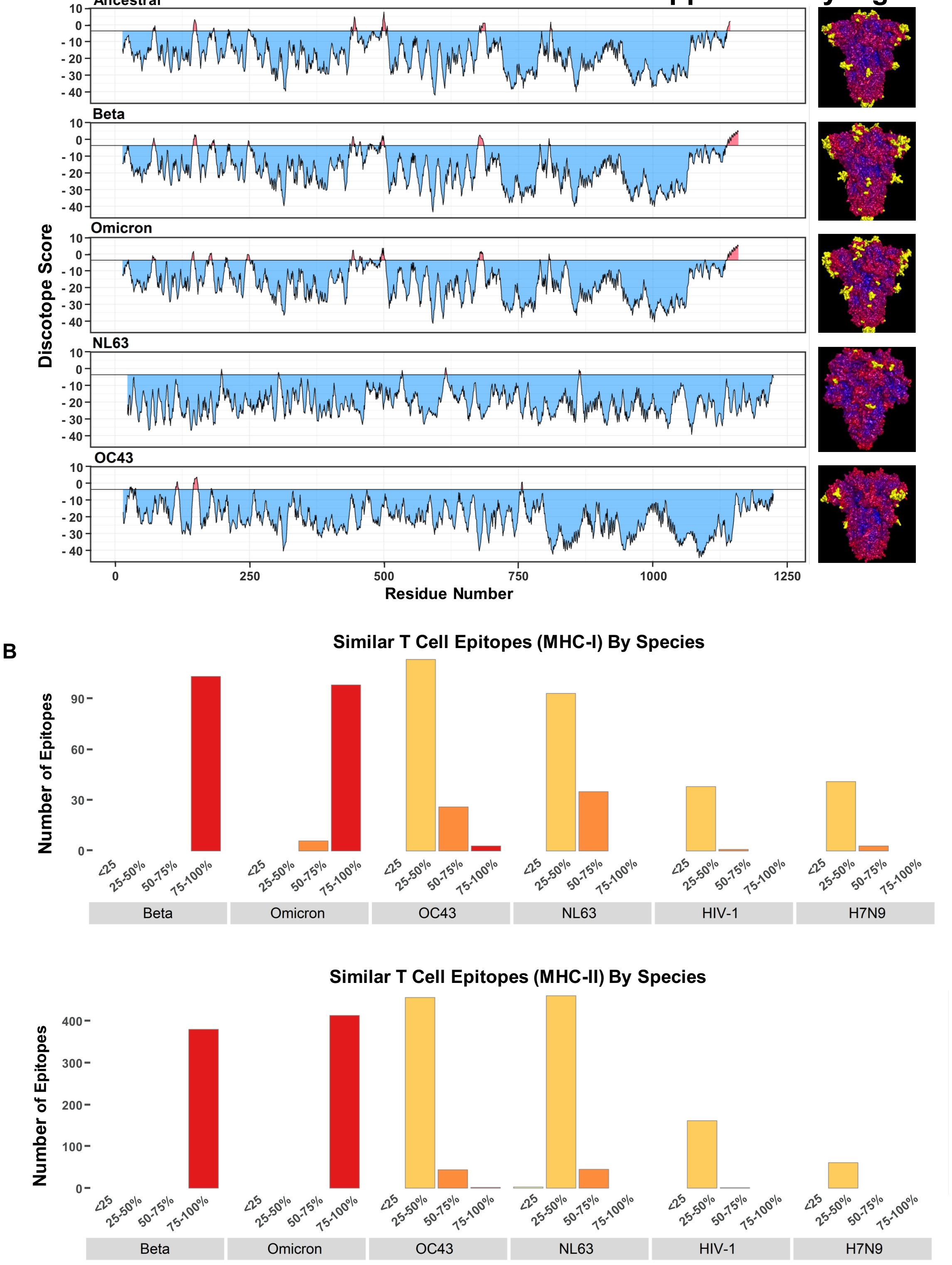

Supplementary Fig. 14. B and T cell epitope analysis among seasonal and SARS-CoV-2 variant coronaviruses reveals less antigenicity in seasonal coronaviruses. The folding models of coronavirus spike proteins were generated with the Phyre2 platform and used to identify surface epitopes using DiscoTope 2.0 (**A**). Results were plotted against the amino acid position (left) as well as in a 3D model predicting epitope position (right). DiscoTope scores falling below (blue) and above (red) the B cell epitope prediction threshold of -3.7 (0.47 sensitivity, 0.75 specificity) were mapped against amino acid sequence number for references ancestral virus, Beta, Omicron, NL63, and OC43. Representative folding models of the spike proteins showing predicted B cell epitopes were made in PyMol and DiscoTope scores are shown as heatmaps along the folded protein, with residues colored according to their predicted score: yellow indicates positively predicted B cell epitopes (scores > -3.7 threshold), red indicates high-scoring amino acids, and blue indicates low scoring regions (i.e., unlikely B cell epitopes). T cell epitopes were predicted using the Immune Epitope Database (IEDB) Analysis Resource NetMHCpan EL 4.1 and MHC II binding was predicted using NetMHCIIpan4.0 EL (**B**). Alleles for MHC class I were selected based on HLA:A and HLA:B variants representative of roughly 97% of the general population. MHC II alleles consisted of HLA-DR, HLA-DQ, and HLA-DP. The percentage of shared epitopes among various coronavirus spike proteins was calculated and binned into one of four percentage brackets: >25%, 25-50%, 50-75%, and 75-100%. The glycoprotein and HA proteins from HIV and influenza virus H7N9 were used as negative controls.

Supplementary Table 1. NL63 Inoculated Hamsters- Statistical Difference from Baseline determined by one-way ANOVA.

Tissue	Nasal Turbinates			Right Cranial Lung		
Day PI	3	6	14	3	6	14
IFN-β	ns	ns	*	ns	ns	ns
STAT2	****	***	*	**	***	ns
IRF1	ns	ns	ns	ns	ns	ns
IRF3	ns	ns	ns	ns	ns	ns
TLR3	****	**	ns	****	*	ns
IFN-γ	ns	ns	ns	ns	*	ns
IRF2	ns	ns	ns	ns	ns	ns
STAT1	ns	*	**	**	ns	ns
CXCL10	****	**	ns	**	ns	ns
CD3	ns	ns	****	ns	ns	ns
CD4	ns	ns	***	ns	ns	***
CD8A	ns	ns	ns	ns	ns	***
IL-2	ns	*	***	*	ns	ns
IL-10	ns	**	ns	ns	ns	ns
CD19	ns	ns	****	ns	ns	*
IL-6	***	ns	ns	****	*	ns
TNF	ns	*	ns	ns	ns	ns
IL-1β	****	**	ns	****	ns	ns

ns: p-value > 0.05
*: p-value = 0.05-0.005
**: p-value = 0.005-0.0005
***: p-value = 0.0005-0.0001
****: p-value < 0.0001

Supplementary Table 2. OC43 Inoculated Hamsters- Statistical Difference from Baseline determined by one-way ANOVA.

Tissue	Nasal Turbinates			Right Cranial Lung		
Day PI	3	6	14	3	6	14
IFN-β	ns	ns	ns	ns	*	*
STAT2	ns	ns	ns	*	ns	ns
IRF1	ns	ns	ns	ns	ns	ns
IRF3	ns	ns	ns	ns	ns	ns
TLR3	ns	*	ns	*	ns	ns
IFN-γ	ns	ns	ns	ns	ns	ns
IRF2	ns	ns	**	ns	ns	ns
STAT1	ns	***	****	ns	ns	ns
CXCL10	****	ns	ns	ns	ns	ns
CD3	ns	ns	****	ns	ns	**
CD4	ns	ns	****	ns	ns	ns
CD8A	ns	**	***	ns	ns	ns
IL-2	ns	***	****	ns	ns	ns
IL-10	ns	****	ns	ns	ns	ns
CD19	ns	ns	****	ns	ns	ns
IL-6	ns	****	ns	***	****	ns
TNF	*	***	ns	ns	ns	ns
IL-1β	****	*	ns	*	ns	*

ns: p-value > 0.05
*: p-value = 0.05-0.005
**: p-value = 0.005-0.0005
***: p-value = 0.0005-0.0001
****: p-value < 0.0001

Supplementary Table 3. Ancestral SARS-CoV-2 Inoculated Hamsters- Statistical Difference from Baseline determined by one-way ANOVA.

Tissue	Nasal Turbinates			Right Cranial Lung		
Day PI	3	6	14	3	6	14
IFN-β	****	ns	ns	****	****	ns
STAT2	ns	ns	ns	**	ns	*
IRF1	ns	ns	ns	ns	*	ns
IRF3	ns	ns	ns	ns	ns	ns
TLR3	ns	ns	ns	ns	ns	ns
IFN-γ	ns	ns	ns	ns	****	ns
IRF2	****	****	ns	****	****	ns
STAT1	****	****	ns	****	****	ns
CXCL10	****	*	ns	ns	****	****
CD3	ns	ns	****	ns	**	****
CD4	ns	*	****	ns	ns	****
CD8A	ns	*	****	ns	ns	****
IL-2	ns	****	ns	ns	****	ns
IL-10	****	****	ns	ns	****	ns
CD19	ns	***	****	ns	ns	****
IL-6	****	****	ns	****	****	ns
TNF	ns	****	ns	****	****	ns
IL-1β	****	****	ns	****	****	ns

ns: p-value > 0.05
*: p-value = 0.05-0.005
**: p-value = 0.005-0.0005
***: p-value = 0.0005-0.0001
****: p-value < 0.0001

Supplementary Table 4. Nasal Turbinates - Statistical Difference from Mock-Ancestral determined by one-way ANOVA.

Primary-Secondary	NL63-Ancestral			OC43-Ancestral			Ancestral-Omicron			Ancestral-Beta			Ancestral-Ancestral		
Day PSI	3	6	14	3	6	14	3	6	14	3	6	14	3	6	14
IFN-β	ns	ns	ns	ns	ns	ns	ns	ns	ns	ns	*	ns	ns	*	ns
STAT2	ns	ns	ns	ns	ns	ns	ns	ns	ns	ns	**	ns	ns	*	ns
IRF1	ns	ns	ns	ns	ns	ns	ns	ns	ns	ns	ns	ns	ns	ns	ns
IRF3	ns	ns	ns	ns	ns	ns	ns	ns	ns	ns	**	ns	*	**	ns
TLR3	ns	ns	ns	ns	ns	ns	ns	ns	ns	ns	***	*	ns	ns	ns
IRF2	ns	ns	ns	ns	ns	ns	ns	ns	ns	ns	ns	ns	ns	ns	ns
STAT1	ns	ns	ns	ns	ns	ns	ns	ns	ns	ns	ns	ns	ns	ns	ns
CXCL10	ns	ns	ns	ns	ns	ns	****	*	ns	****	***	ns	****	ns	ns
IFN-γ	ns	ns	ns	ns	ns	ns	ns	ns	ns	ns	ns	ns	ns	ns	ns
IL-12	ns	ns	ns	**	ns	ns	ns	ns	**	ns	***	ns	ns	**	ns
T-bet	ns	ns	ns	*	***	ns	*	**	ns	**	****	ns	***	****	ns
CD3	ns	ns	ns	ns	ns	ns	ns	ns	****	ns	ns	****	ns	ns	****
IL-2	ns	ns	ns	ns	ns	ns	ns	ns	ns	ns	**	ns	ns	ns	ns
IL-21	ns	ns	ns	ns	ns	ns	ns	****	ns	ns	****	ns	ns	****	ns
CXCR5	ns	ns	ns	*	**	*	***	ns	*	****	****	*	****	***	*
CD4	ns	ns	ns	ns	***	***	ns	ns	ns	ns	ns	ns	ns	ns	*
CD8A	ns	ns	ns	ns	ns	ns	ns	ns	****	ns	*	****	**	ns	****
PRF1	ns	ns	ns	ns	ns	ns	****	ns	ns	**	ns	ns	****	ns	ns
GZMB	ns	ns	ns	**	*	ns	****	ns	ns	****	*	ns	****	*	ns
GATA-3	ns	ns	ns	ns	ns	ns	**	**	****	**	**	****	***	****	****
IL-4	ns	ns	ns	ns	ns	ns	*	*	**	ns	ns	ns	ns	ns	ns
IL-5	ns	ns	ns	ns	ns	ns	****	****	ns	ns	***	**	**	***	ns
IL-13	ns	ns	ns	ns	ns	ns	****	****	ns	ns	****	*	ns	***	ns
ROR-γ-T	ns	ns	ns	*	ns	ns	*	ns	ns	ns	*	ns	ns	ns	ns
IL-17	ns	ns	ns	*	ns	ns	****	ns	ns	****	ns	ns	****	ns	ns
IL-22	ns	ns	ns	****	ns	ns	ns	ns	ns	ns	ns	ns	ns	ns	ns
FoxP3	ns	ns	ns	ns	ns	ns	ns	ns	ns	***	***	*	*	***	*
IL-10	ns	ns	ns	ns	ns	ns	****	**	ns	****	ns	****	****	****	ns
TGF-β	ns	ns	ns	ns	ns	ns	ns	ns	ns	ns	ns	ns	ns	ns	ns
CD19	ns	ns	ns	ns	ns	ns	ns	****	ns	**	ns	***	ns	ns	ns
AID	ns	ns	ns	ns	ns	ns	***	ns	ns	***	**	ns	***	ns	ns
BCL6	ns	***	ns	ns	ns	ns	*	****	ns	*	***	ns	***	**	ns
IL-6	ns	ns	ns	ns	ns	ns	****	****	ns	****	****	ns	****	****	ns
IL-1β	****	ns	ns	****	ns	ns	ns	ns	ns	****	ns	ns	****	*	ns
TNF	ns	ns	ns	ns	ns	ns	ns	*	ns	ns	*	ns	ns	***	ns

ns: p-value > 0.05
*: p-value = 0.05-0.005
**: p-value = 0.005-0.0005
***: p-value = 0.0005-0.0001
****: p-value < 0.0001

Supplementary Table 5. Right Cranial Lung - Statistical Difference from Mock-Ancestral determined by one-way ANOVA.

Primary-Secondary	NL63-Ancestral			OC43-Ancestral			Ancestral-Omicron			Ancestral-Beta			Ancestral-Ancestral		
Day PSI	3	6	14	3	6	14	3	6	14	3	6	14	3	6	14
IFN-β	ns	ns	ns	ns	ns	ns	****	**	ns	***	****	****	****	****	ns
STAT2	ns	ns	ns	ns	ns	ns	ns	*	ns	ns	*	ns	ns	*	ns
IRF1	ns	ns	ns	ns	ns	ns	ns	ns	ns	ns	ns	ns	ns	ns	ns
IRF3	ns	ns	ns	ns	ns	ns	ns	*	ns	*	*	ns	*	*	ns
TLR3	ns	ns	ns	ns	ns	ns	ns	ns	ns	ns	ns	ns	*	ns	ns
IRF2	*	ns	*	*	ns	ns	*	*	ns	*	**	ns	*	*	ns
STAT1	****	ns	ns	****	ns	ns	***	ns	ns	****	ns	ns	****	ns	ns
CXCL10	ns	ns	ns	ns	ns	ns	****	*	ns	ns	ns	ns	ns	ns	ns
IFN-γ	ns	ns	ns	ns	ns	ns	**	****	ns	ns	****	ns	ns	****	ns
IL-12	ns	ns	ns	ns	****	ns	ns	ns	ns	ns	ns	*	ns	***	ns
T-bet	ns	ns	ns	ns	ns	ns	**	ns	*	ns	ns	**	*	ns	**
CD3	ns	ns	ns	ns	ns	ns	***	*	ns	***	****	*	**	****	****
IL-2	ns	ns	ns	ns	****	ns	ns	ns	ns	****	ns	*	**	ns	ns
IL-21	ns	ns	ns	ns	*	*	ns	****	*	ns	****	****	ns	****	****
CXCR5	ns	ns	ns	ns	ns	ns	*	ns	*	ns	ns	*	**	**	**
CD4	ns	ns	ns	ns	ns	ns	ns	***	ns	****	ns	*	***	ns	***
CD8A	ns	ns	ns	ns	ns	ns	ns	ns	ns	**	***	***	*	*	***
PRF1	ns	ns	ns	*	ns	ns	***	ns	ns	***	ns	ns	***	ns	ns
GZMB	ns	ns	ns	*	ns	ns	****	ns	ns	****	ns	ns	***	ns	ns
GATA-3	ns	ns	ns	ns	ns	ns	ns	ns	ns	ns	ns	ns	ns	ns	ns
IL-4	ns	ns	ns	ns	ns	ns	ns	****	ns	ns	ns	ns	ns	****	ns
IL-5	ns	ns	ns	ns	ns	ns	ns	ns	****	****	ns	****	**	ns	ns
IL-13	ns	ns	ns	ns	ns	ns	ns	*	ns	**	ns	ns	ns	ns	ns
ROR-γ-T	ns	ns	ns	ns	ns	ns	***	ns	ns	***	ns	ns	*	ns	ns
IL-17	ns	ns	ns	***	ns	ns	ns	**	ns	ns	**	ns	ns	**	ns
IL-22	ns	ns	ns	ns	ns	ns	ns	ns	ns	ns	ns	ns	ns	ns	ns
FoxP3	ns	ns	ns	ns	ns	ns	ns	**	ns	**	ns	ns	**	ns	ns
IL-10	ns	ns	ns	ns	ns	ns	ns	**	ns	****	****	****	ns	ns	****
TGF-β	ns	*	ns	ns	ns	ns	ns	ns	**	ns	ns	ns	ns	*	ns
CD19	ns	ns	ns	ns	ns	ns	ns	ns	***	*	***	ns	ns	ns	***
AID	*	**	ns	ns	ns	ns	*	*	ns	ns	ns	ns	***	*	ns
BCL6	*	**	ns	ns	ns	ns	ns	ns	ns	ns	ns	ns	**	ns	ns
IL-6	ns	ns	ns	ns	*	ns	****	ns	ns	ns	****	ns	ns	***	*
IL-1β	*	ns	ns	ns	ns	ns	ns	**	****	*	***	*	*	**	*
TNF	***	ns	ns	***	ns	ns	***	****	ns	****	****	**	****	****	ns

ns: p-value > 0.05
*: p-value = 0.05-0.005
**: p-value = 0.005-0.0005
***: p-value = 0.0005-0.0001
****: p-value < 0.0001

Supplementary Table 6. Mediastinal Lymph Node – Statistical Difference from Mock-Ancestral determined by one-way ANOVA.

Primary-Secondary	NL63-Ancestral			OC43-Ancestral			Ancestral-Omicron			Ancestral-Beta			Ancestral-Ancestral		
Day PSI	3	6	14	3	6	14	3	6	14	3	6	14	3	6	14
IFN-β	ns	ns	ns	ns	ns	ns	ns	ns	ns	ns	ns	**	ns	ns	ns
STAT2	ns	ns	ns	ns	ns	ns	ns	ns	ns	ns	ns	ns	ns	ns	ns
IRF1	ns	ns	**	ns	ns	ns	ns	*	ns	ns	ns	ns	ns	**	ns
IRF3	***	ns	ns	ns	ns	ns	ns	ns	ns	ns	ns	ns	ns	ns	ns
TLR3	ns	ns	ns	ns	ns	ns	ns	ns	*	ns	ns	ns	ns	ns	**
IRF2	**	ns	ns	*	ns	ns	*	**	ns	**	**	ns	**	**	**
STAT1	ns	ns	ns	ns	ns	ns	**	*	ns	**	**	ns	**	**	**
CXCL10	ns	ns	ns	ns	ns	ns	ns	ns	**	ns	**	*	ns	**	**
IFN-γ	ns	ns	ns	ns	*	ns	ns	*	ns	**	**	****	***	**	****
IL-12	ns	ns	ns	*	ns	ns	*	ns	ns	ns	*	*	ns	*	*
T-bet	**	****	ns	***	*	ns	**	***	****	***	***	****	***	****	****
CD3	ns	ns	ns	ns	ns	ns	ns	ns	ns	ns	*	ns	ns	****	*
IL-2	ns	ns	ns	**	ns	ns	ns	ns	ns	ns	*	****	ns	*	****
IL-21	ns	****	****	ns	**	****	ns	****	****	ns	****	****	***	****	**
CXCR5	ns	ns	ns	*	*	*	***	***	**	****	****	****	****	****	****
CD4	ns	ns	ns	ns	ns	ns	ns	ns	ns	ns	ns	**	ns	ns	****
CD8A	**	ns	*	ns	ns	ns	ns	ns	***	ns	ns	****	ns	ns	****
PRF1	ns	ns	ns	ns	ns	ns	ns	ns	ns	ns	ns	ns	ns	ns	ns
GZMB	ns	ns	ns	**	ns	ns	**	ns	ns	*	ns	ns	*	ns	ns
GATA-3	ns	ns	ns	ns	ns	ns	***	ns	ns	****	*	ns	****	*	ns
IL-4	ns	ns	ns	ns	ns	ns	ns	*	**	**	*	****	**	ns	*
IL-5	ns	ns	ns	ns	ns	ns	*	**	**	**	**	**	**	***	ns
IL-13	ns	ns	ns	ns	ns	ns	*	ns	**	*	ns	***	****	ns	****
ROR-γ-T	ns	ns	ns	ns	ns	**	ns	*	**	ns	ns	ns	ns	ns	ns
IL-17	ns	ns	ns	ns	ns	ns	ns	ns	ns	ns	ns	ns	ns	ns	ns
IL-22	ns	ns	ns	ns	ns	ns	ns	ns	ns	ns	****	***	***	ns	***
FoxP3	ns	ns	*	ns	ns	*	ns	**	****	ns	****	*	ns	ns	****
IL-10	ns	*	*	ns	ns	*	ns	**	****	ns	*	**	ns	**	ns
TGF-β	ns	ns	ns	ns	ns	ns	****	*	ns	ns	ns	ns	ns	*	ns
CD19	***	****	ns	ns	ns	ns	ns	****	**	ns	****	***	**	****	ns
AID	ns	ns	ns	ns	ns	ns	*	ns	ns	****	***	*	****	**	ns
BCL6	ns	ns	ns	ns	ns	ns	ns	**	ns	****	****	ns	****	****	ns
IL-6	ns	ns	ns	ns	ns	ns	ns	ns	ns	ns	****	****	ns	****	***
IL-1β	ns	ns	ns	ns	ns	ns	ns	ns	ns	ns	ns	****	***	***	****
TNF	ns	ns	**	ns	ns	ns	ns	ns	ns	ns	ns	****	ns	ns	****

ns: p-value > 0.05
*: p-value = 0.05-0.005
**: p-value = 0.005-0.0005
***: p-value = 0.0005-0.0001
****: p-value < 0.0001

Fig. 7C
