# Supplementary material for: Previous infection with seasonal coronaviruses does not protect male Syrian hamsters from challenge with SARS-CoV-2
Source: Nat Commun. 2023 Sep 26;14:5990. doi: 10.1038/s41467-023-41761-1 (PMC10522707; doi:10.1038/s41467-023-41761-1)
Supplement: Supplementary file 2 — Reporting Summary [file 41467_2023_41761_MOESM2_ESM.pdf]

nature portfolio

Corresponding author(s): Dr. Alyson Kelvin

Last updated by author(s): Aug 17, 2023

Reporting Summary

Nature Portfolio wishes to improve the reproducibility of the work that we publish. This form provides structure for consistency and transparency in reporting. For further information on Nature Portfolio policies, see our Editorial Policies and the Editorial Policy Checklist.

Statistics

For all statistical analyses, confirm that the following items are present in the figure legend, table legend, main text, or Methods section.

n/a	Confirmed	
☐	☒	The exact sample size (*n*) for each experimental group/condition, given as a discrete number and unit of measurement
☐	☒	A statement on whether measurements were taken from distinct samples or whether the same sample was measured repeatedly
☐	☒	The statistical test(s) used AND whether they are one- or two-sided<br>*Only common tests should be described solely by name; describe more complex techniques in the Methods section.*
☐	☒	A description of all covariates tested
☐	☒	A description of any assumptions or corrections, such as tests of normality and adjustment for multiple comparisons
☐	☒	A full description of the statistical parameters including central tendency (e.g. means) or other basic estimates (e.g. regression coefficient) AND variation (e.g. standard deviation) or associated estimates of uncertainty (e.g. confidence intervals)
☐	☒	For null hypothesis testing, the test statistic (e.g. *F*, *t*, *r*) with confidence intervals, effect sizes, degrees of freedom and *P* value noted<br>*Give P values as exact values whenever suitable.*
☒	☐	For Bayesian analysis, information on the choice of priors and Markov chain Monte Carlo settings
☒	☐	For hierarchical and complex designs, identification of the appropriate level for tests and full reporting of outcomes
☒	☐	Estimates of effect sizes (e.g. Cohen's *d*, Pearson's *r*), indicating how they were calculated

Our web collection on statistics for biologists contains articles on many of the points above.

Software and code

Policy information about availability of computer code

Data collection	All data collected from databases are publicly available and did not inolve custom algorithms or novel software. Raw sequences of viral strains were collected from GISAID for viral identity analysis. S-protein reference sequences were collected from GISAID or NCBI for MHC I and MIHC II binding predictions.
Data analysis	All data analysis utlized publicly available software. No custom code was generated. BLOcks SUbstitution Matrices (BLOSUM) was used for viral identity analysis. Viral sequences were imported into Geneious35 Prime (2022.2.2) as a FASTA file and sequences were selected to perform pairwise alignment using Geneious Alignment. Parameters were set to global alignment with free end gaps at cost matrix BLOSUM90 and a gap open penalty and extension penalty at 12 and 3 respectively. S-protein reference sequences were screened for predicted MHC-I compatible peptides through the NetMHCpan EL 4.1 program. MHC -II binding was predicted using NetMHCII 2.3 nn-align. Predicted MHC compatible peptides were compared to SARS CoV-2 Wuhan using the Epitope conservancy Analysis tool (IEDB).

For manuscripts utilizing custom algorithms or software that are central to the research but not yet described in published literature, software must be made available to editors and reviewers. We strongly encourage code deposition in a community repository (e.g. GitHub). See the Nature Portfolio guidelines for submitting code & software for further information.

Data

Policy information about availability of data

All manuscripts must include a data availability statement. This statement should provide the following information, where applicable:

- Accession codes, unique identifiers, or web links for publicly available datasets
- A description of any restrictions on data availability
- For clinical datasets or third party data, please ensure that the statement adheres to our policy

All accession codes, publicly available datasets and data generated and analyzed during this study are included in this published article and its Supplementary Information files.

Human research participants

Policy information about studies involving human research participants and Sex and Gender in Research.

Reporting on sex and gender	No human research participants were involved in the study.
Population characteristics	N/A
Recruitment	N/A
Ethics oversight	N/A

Note that full information on the approval of the study protocol must also be provided in the manuscript.

Field-specific reporting

Please select the one below that is the best fit for your research. If you are not sure, read the appropriate sections before making your selection.

☒ Life sciences ☐ Behavioural & social sciences ☐ Ecological, evolutionary & environmental sciences

For a reference copy of the document with all sections, see nature.com/documents/nr-reporting-summary-flat.pdf

Life sciences study design

All studies must disclose on these points even when the disclosure is negative.

Sample size	Sample size was determined based on precedent set in the field by our research group (Francis et al., 2021) and others (Imai et al., 2020). Following these previous reports, we utilized three animals per time point, per group, for sample collection and a minimum of six animals per group for survival and weight loss.
Data exclusions	No data were excluded from the analysis.
Replication	All clinical data included sufficient animals numbers to ensure repeatability. Select infections were repeated 2-3 times to ensure reproducibility. All viral load analysis, histology assessment and host gene expression analysis is assessed in triplicate and neutralization assays are run in duplicate. All attempts at replication were successful.
Randomization	Allocation of animals to experimental groups was random.
Blinding	Investigators were blinded to group allocation during data collection and analysis.

Reporting for specific materials, systems and methods

We require information from authors about some types of materials, experimental systems and methods used in many studies. Here, indicate whether each material, system or method listed is relevant to your study. If you are not sure if a list item applies to your research, read the appropriate section before selecting a response.

Materials & experimental systems

n/a	Involved in the study
☒	☐ Antibodies
☐	☒ Eukaryotic cell lines
☒	☐ Palaeontology and archaeology
☐	☒ Animals and other organisms
☒	☐ Clinical data
☒	☐ Dual use research of concern

Methods

n/a	Involved in the study
☒	☐ ChIP-seq
☒	☐ Flow cytometry
☒	☐ MRI-based neuroimaging

Eukaryotic cell lines

Policy information about cell lines and Sex and Gender in Research

Cell line source(s)	Vero 76 Cells (ATCC CRL-1587); Vero Cells (ATCC CCL-81); LLC-MK2 Cells (ATCC CCL-7); HCT-8 Cells (ATCC CCL-244); MRC-5 Cells (ATCC CCL-171)
Authentication	Cell lines were purchased directly from ATCC. Cell morphology is evaluated on a weekly basis and records are kept for expansions, storage, and passage.
Mycoplasma contamination	Cell lines are monitored monthly for mycoplasma using InvivoGen's MycoStrip Mycoplasma Detection Kit (Product Code: rep-mys).
Commonly misidentified lines (See ICLAC register)	No cell lines used are registered as commonly misidentified cell lines.

Animals and other research organisms

Policy information about studies involving animals; ARRIVE guidelines recommended for reporting animal research, and Sex and Gender in Research

Laboratory animals	LVG Golden Syrian Hamster (Charles River Laboratories), 8-weeks old
Wild animals	The study did not involve the use of wild animals.
Reporting on sex	Only male hamsters were utilized in this study. 108 animals were used in this study. Female hamsters were not utilized as they are aggressive towards one another when co-housed and containment level 3 limitations on housing animals would not permit the use of female animals. We therefore do not make conclusions based on the sex of these animals.
Field-collected samples	The study did not involve samples collected from the field.
Ethics oversight	All work was conducted in accordance with the Canadian Council of Animal Care (CCAC) guidelines, AUP number 20200019 by the University Animal Care Committee (UACC) Animal Research Ethics Board at the University of Saskatchewan. This article is published with the permission of the Director of Vaccine and Infectious disease organization (VIDO) manuscript no. 1012.

Note that full information on the approval of the study protocol must also be provided in the manuscript.
